# Recovery of olfactory function after nine years of post-traumatic anosmia: a case report

**DOI:** 10.4076/1752-1947-3-9283

**Published:** 2009-09-16

**Authors:** Christian A Mueller, Thomas Hummel

**Affiliations:** 1 Department of Otorhinolaryngology, Medical University Vienna, Waehringer Guertel 18-20, 1090 Wien, Austria; 2 Smell & Taste Clinic, Department of Otorhinolaryngology, University of Dresden Medical School, Fetscherstr. 74, 01307 Dresden, Germany

## Abstract

**Introduction:**

Olfactory loss due to head trauma is a common condition. Depending on the severity of the head trauma, anosmia might occur in up to 30% of patients. The period of time until recovery has been reported to be a couple of months in most cases. However, recovery from post-traumatic olfactory loss might occur much later. We present a rare case of recovery from anosmia nine years after the initial trauma.

**Case presentation:**

We report the case of a 54-year-old Caucasian man who suffered complete anosmia from a severe car accident. Smell function as well as flavor perception during eating and drinking were also completely lost. After nine years, the patient had his first olfactory impressions, with his sense of smell gradually improving over a period of three years. We confirmed recovery of olfactory function using psychophysical and electrophysiological techniques.

**Conclusion:**

In most cases, recovery of smell function occurs relatively soon after the head trauma and seems to rarely occur more than two years after the incident. However, patients should be informed that there is a small chance of recovery a long time after the trauma.

## Introduction

Approximately 5% to 20% of all patients presenting themselves to specialized centers with olfactory dysfunction are diagnosed with post-traumatic disorders. Together with post-infectious olfactory loss and sinunasal disease, head trauma is the most common cause of smell impairment [1,2]. Depending on the severity of the head trauma, anosmia might occur in up to 30% of cases [3].

The level of recovery from post-traumatic olfactory loss was found to be approximately 10% [4,5]. In most cases, it takes a few months until first olfactory impressions are reported [6]. However, recovery from post-traumatic olfactory loss has been recorded five years [6] and seven years [4] after olfactory loss.

Late recovery from anosmia due to head trauma is believed to occur because of regeneration of olfactory nerve fibers and their reconnection with central neurons of the olfactory bulb. This mechanism was shown in hamsters after transsection of the olfactory nerves [7]. In humans, reconnection might be prevented in most cases by mechanical occlusion of the cribriform plate with fibrotic tissue. In this report, we present a rare case of recovery from anosmia after nine years. Recovery of olfactory function was confirmed using psychophysical and electrophysiological techniques.

## Case presentation

Our 54-year-old male Caucasian patient had had a severe car accident at the age of 38. He suffered multiple fractures of the central face. His right eye had to be enucleated and replaced by a prosthesis. The patient underwent multiple surgeries and stayed in the intensive care unit for more than two weeks, and then for several weeks in the hospital. He noticed a complete loss of his sense of smell, which was confirmed in subsequent litigation. The patient reported that he was not able to smell smoke or gas and he could not detect flavor in food and beverages. Consequently, the patient's quality-of-life significantly decreased.

Approximately nine years after the accident, he reported his first olfactory impression. It was the smell of hay which was perceived during a walk. The ability to smell continually improved over three years, and has stayed constant since then. No specific therapies regarding smell function were given to the patient. Today, he has no problems with his sense of smell and has normal flavor perception during eating and drinking.

**Figure 1:**
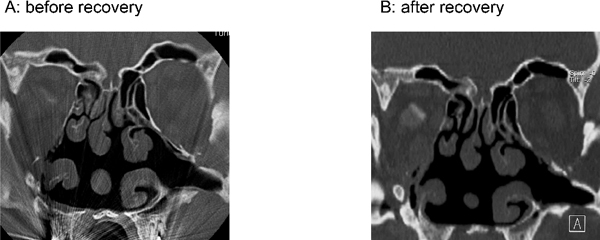
**Computed tomography scans before and after recovery of olfactory function**. Both scans (before **[A, left]** and after **[B, right]** recovery from post-traumatic anosmia) show the same anatomical structures. There was no sign of either conductive olfactory loss due to obstruction of the olfactory cleft, or sinunasal disease.

Computed tomography (Figure 1) showed patent olfactory clefts, both after the accident, when the patient was anosmic, and 10 years later, when he had regained his olfactory abilities. These images ruled out the presence of obstructions due to sinunasal disease as possible causes of smell dysfunction.

After complete ear-nose-throat examination including nasal endoscopy, smell function was tested 16 years after the accident using the 'Sniffin' Sticks' test battery [8]. This test has been extensively validated and comprises three subtests: a test of olfactory threshold, an odor discrimination task, and an odor identification test. The patient yielded 28.5 points, which represents a score within the lower normal range [9].

**Figure 2:**
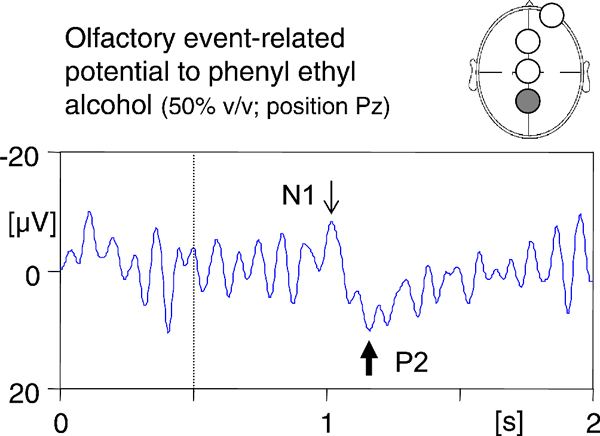
**Olfactory event-related potential at recording position Pz (see filled circle on schematic drawing in inset) in response to the olfactory stimulant phenylethyl alcohol (rose-like odor; 50% v/v; interstimulus interval 50 seconds; onset of stimuli of 200 ms duration marked with dotted line)**. The two major peaks of the olfactory event-related potential (N1 and P2) are indicated with thin and thick arrows, respectively.

In order to confirm the absence of anosmia, evoked-response olfactometry was applied to the patient [10] using an olfactometer (Burghart Instruments, Wedel, Germany). Following stimulation with selective olfactory stimuli (50% v/v phenylethanol), event-related potentials were clearly detectable (Figure 2).

## Discussion

Our patient's history suggests post-traumatic olfactory loss, possibly due to shearing of olfactory nerve fibers passing through the ethmoidal cribriform plate. Theoretically, it might also be due to contusion of the olfactory bulb and/or frontal lobe [11].

Histological studies of olfactory mucosa from patients with post-traumatic anosmia have demonstrated extensive axonal regeneration near the basal membrane. Moreover, changes in the epithelial architecture and loss of peripheral cilia have been found [12].

In most cases, therapeutic options are lacking in patients with post-traumatic anosmia although systemic corticosteroids are used to reduce possible edema of the central regions [2]. Patients suffering from post-traumatic smell disorders should be informed about possible hazardous events due to their disability. These may include cooking accidents, the failure to detect smoke or gas, as well as eating spoiled foods. A recently published study found that patients with olfactory disorders are at least at double the risk of suffering from one of these potentially life-threatening events [13]. As pointed out above, in most cases, recovery of smell function after head trauma occurs soon after the accident. Recovery after more than one or two years seems to be relatively rare. In terms of medico-legal cases, this means that, after this period of time, the diagnosis of post-traumatic anosmia can be regarded as definite. However, patients should be informed that a certain possibility of recovery exists even after a long period of time, although the exact mechanism still remains unclear.

## Consent

Written informed consent was obtained from the patient for publication of this case report and any accompanying images. A copy of the written consent is available for review by the Editor-in-Chief of this journal.

## Competing interests

The authors declare that they have no competing interests.

## Authors' contributions

CAM investigated the patient, performed the smell tests and drafted the manuscript. TH prepared the figures after analysis and interpretation of the data obtained by evoked-response olfactometry and revised the manuscript. Both authors read and approved the final manuscript.
